# Reoperation for Recurrent Adrenocortical Carcinoma: A Systematic Review and Pooled Analysis of Population-Based Studies

**DOI:** 10.3389/fsurg.2022.781406

**Published:** 2022-02-17

**Authors:** Fan Zhang, Zhihong Liu, Dechao Feng, Yongquan Tang, Shenzhuo Liu, Kan Wu, Fuxun Zhang, Yuchun Zhu, Yiping Lu

**Affiliations:** Department of Urology/Institute of Urology, West China Hospital, Sichuan University, Chengdu, China

**Keywords:** adrenocortical carcinoma, reoperation, recurrence, meta-analysis, system review

## Abstract

**Background:**

Adrenocortical carcinoma (ACC) is a rare neoplasm with a high recurrence rate. This study aimed to assess the role of surgery in the clinical management of recurrent ACC.

**Methods:**

The PubMed, Embase, Web of Science, and Cochrane Library databases were searched, and the hazard ratios were pooled.

**Results:**

Patients who underwent resection for recurrence had significantly better OS or OS after recurrence than those who received only nonsurgical treatments (HR 0.34, *p* < 0.001). Prognostic factors were associated with decreased OS after recurrence, including multiple recurrence (HR 3.23, *p* = 0.001), shorter disease-free interval (HR 2.94, *p* < 0.001), stage III-IV of the original tumor (HR 6.17, *p* = 0.001), sex of male (HR 1.35, *p* = 0.04), and initial non-R0 resection (HR 2.13, *p* = 0.001). Prolonged OS after recurrence was observed in those who experienced incomplete resection (HR 0.43, 95% CI 0.31–0.52, I^2^ = 53%) compared with patients who only received nonsurgical treatments. In the reoperated group, patients who underwent complete resection of recurrence had a prolonged OS after recurrence compared with those who underwent incomplete resection (HR 0.23, *p* = 0.004).

**Conclusions:**

We confirmed the role of reoperation in the clinical management of recurrent ACC. Select patients might benefit from debulking surgery. The preoperative evaluation of the complete resection of the recurrence is the key means to decide whether patients should undergo surgery. Other prognostic factors associated with prolonged OS include single recurrence site, relatively longer disease-free interval, stage I-II of the original tumor, and female sex.

## Introduction

Adrenocortical carcinoma (ACC) is a rare endocrine neoplasm with an estimated annual incidence of 1–2 per million inhabitants and has one of the poorest prognoses (1, 2). Patients are frequently asymptomatic, and most tumors are discovered at an advanced stage by symptoms of mass effects (3). Patients are usually diagnosed with an invasion of adjacent organs or metastatic disease, and the prognosis of ACC is extremely poor. For patients with stage I–II of the disease, the 5-year survival rate is ~60% (4), whereas it is 40 and 28% for patients with stage III and IV disease, respectively (5).

However, evidence shows that patients with ACC rarely benefit from chemotherapy and radiation therapy. Complete tumor resection remains the only curative treatment for ACC. Although complete tumor resection has been performed, as many as 74% of patients experience local recurrence or distant metastasis (6). In recent decades, progress has been made in the clinical management of ACC patients using mitotane. However, for managing patients who undergo recurrent ACC, therapeutic options are still strictly limited. Prognostic factors of recurrent ACC have not yet been firmly established. Some recent studies suggested a benefit with survival in recurrent ACC patients undergoing reoperation (7, 8). However, whether we should take the very aggressive resection approach for patients with advanced disease is still controversial. The value of surgical treatment, especially when it requires repeated or debulking surgeries, remains to be confirmed. Therefore, we systematically pooled previous studies to assess the role of reoperation in the clinical management of recurrent ACC and attempted to identify the clinical characteristics of patients who can benefit from reoperation.

## Methods

### Data Source

A systematic review was carried out following the Preferred Reporting Items for Systematic Reviews and Meta-Analyses (PRISMA) statement (9). The PubMed, Embase, Web of Science, and Cochrane Library databases were comprehensively searched between January 1990 and December 2020. The OVID tool was used to retrieve the Embase and Cochrane libraries. The following mesh terms were used: “adrenocortical carcinoma,” “surgery,” and “recurrence”. In addition, we manually retrieved the related studies from the reference of retrieved studies in case of missing data.

### Inclusion and Exclusion Criteria

Studies were considered eligible for final analysis if they: (1) were a population-based study; (2) involved patients with recurrent ACC; (3) compared reoperation with nonsurgical therapy, or identified the clinical characteristics of patients who can benefit more from reoperation; (4) used survival analysis to report relevant clinical outcomes; and (5) included sufficient data for analyses. Studies were excluded if they: (1) were in a non-English language; (2) did not distinguish recurrent ACC patients from metastatic ACC patients; (3) did not distinguish recurrent ACC patients from patients without recurrence (Studies were excluded from this research when they did not distinguish patients who did not undergo primary resection before metastasectomy from patients who underwent reoperation after the resection of primary tumor); (4) were not available or were published before 1990; (5) contained insufficient data for analyses; and (6) were review, cases, or conference abstracts. The titles and abstracts of all retrieved studies were screened by two independent investigators (ZF and FDC) to exclude irrelevant studies. Independent assessment was then conducted in duplicate for the full texts of potentially relevant studies. Any differences and conflicts were resolved through discussion and consensus by the group. For records containing the same population, the data of the latest study were extracted.

### Data Extraction and Quality Assessment

The following relevant information was extracted from each eligible study: (1) publication details, first author's name, publication year, enrollment data and location; (2) age, number, and follow-u*p* time of patients, clinical outcomes; and (3) hazard ratios (HRs), 95% confidence intervals (CIs) of reoperation and other clinical parameters mentioned below for overall survival (OS). If only Kaplan–Meier curves were available, then we used Engauge Digitizer version 12.1 (http://digitizer.sourceforge.net/) to extract data, and HRs and 95% CIs were then calculated using the described method (10–12). For studies containing both univariate and multivariate analyses, we prioritized the multivariate analysis results with the promise of eliminating the influence of other confounding factors. The Newcastle–Ottawa Quality Assessment Scale (NOS) was used (13) to evaluate the quality of retrospective studies, including three parameters: the selection parameter, the comparability parameter, and the outcome parameter. Studies with a score > 6 were regarded as high quality. Stage classification was based on the European Network for the Study of Adrenal Tumors (ENSAT) or the American Joint Committee on Cancer (AJCC) (14, 15).

### Statistical Analysis

We used HRs with 95% CIs to assess the correlation between clinical parameters and outcomes of recurrent ACC patients. Further analysis was applied in patients who underwent reoperation to evaluate which patients would benefit more from reoperation. RevMan 5.3 software was used to calculate a summary hazard ratio or to indicate the association between clinical parameters and mortality. We used Cochran's Q test and Higgins I squared (I2) statistic to examine the heterogeneity between the studies. *P* > 0.1 or I2 < 50% was identified as no or moderate heterogeneity, and the fixed-effect model was applied; otherwise, the random-effects model was used (16). In addition, sensitivity analysis was conducted to identify the stability of the results. We also evaluated publication bias among the included studies based on Begg's test. *P* < 0.05 was regarded as statistically significant.

## Results

### Identification and Selection of Included Studies

**Figure 1:**
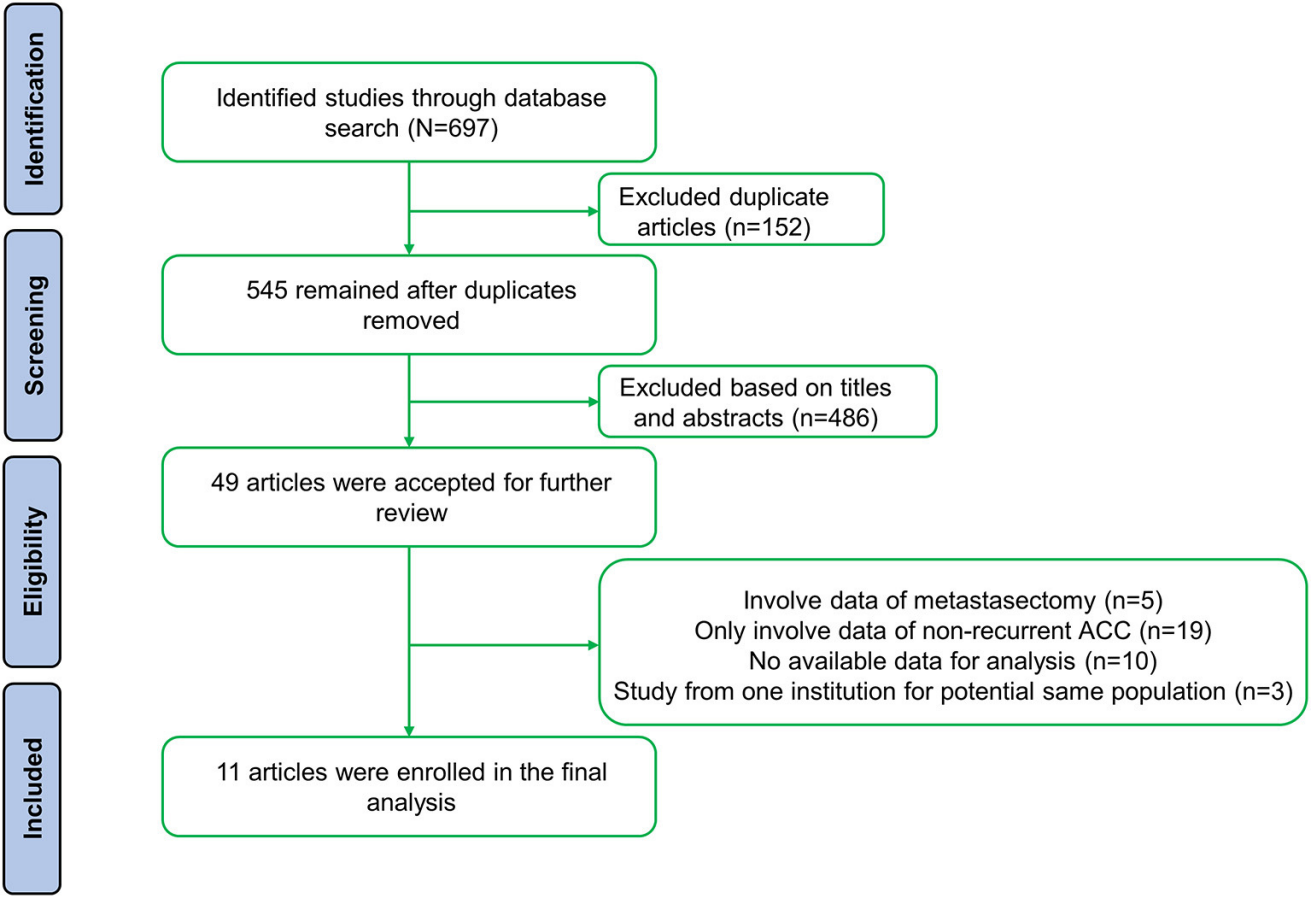
Flow chart of the study search and selection.

A total of 697 studies were retrieved based on the described criteria. After removing 152 duplicate records, 545 articles were collected for preliminary screening of titles and abstracts. Then, we initially identified 49 potentially relevant papers and screened the full text. Ultimately, after excluding 37 studies [19 that only involved data on nonrecurrent ACC, 10 that did not have available data for analysis, five that contained patients who underwent metastasectomy without recurrence (17–21), and three that included the same population (21–23)], 11 retrospective studies including 964 patients from five countries were involved (Figure 1) (7, 8, 15, 24–31). Nine studies divided patients into reoperated and nonreoperated groups, while five studies reported the results of reoperated patients to identify prognostic factors that were related to improved OS for repeated surgeries (Table 1). Of the included studies, reoperation was performed in 573 patients, whereas 391 received only nonsurgical treatments after recurrence.

**Table 1:** Clinical characteristics of the included studies.

**References**	**Enrollment date, location**	**Study type**	**Treatment**	**No. of patients (reoperated vs. nontreated)**	**Follow-up, months**	**Nos**	**OS calculation**
Simon et al. (7)	1980–2014, France	Retrospective	Reoperation vs. no surgery	59 (29 vs.30)	Median 69 (range 7–148)	8	After recurrence
Erdogan et al. (29)	Since 2003, Germany	Retrospective	Reoperation vs. no surgery	154 (101 vs. 53)	Median 69 (range 30-297)	7	After recurrence
Glenn et al. (8)	1983–2017, USA	Retrospective	Reoperation vs. no surgery	242 (100 vs. 142)	Median 35 (range 0.2-295)	8	Overall
Dy et al. (28)	1980–2010, USA	Retrospective	Reoperation vs. no surgery	93 (67 vs. 26)	Median 48 (range 2-239)	7	After recurrence
Bellantone et al. (25)	Not reported, Italy	Retrospective	Reoperation vs. no surgery	52 (20 vs. 32)	Median 21.7	7	Overall
Tran et al. (15)	1997–2014. USA	Retrospective	Reoperation	56	Not reported	8	After reoperation
Jensen et al. (24)	1965–1989, USA	Retrospective	Reoperation vs. no surgery	33 (15 vs. 18)	Not reported	7	After recurrence
Gonzalez et al. (27)	1991–2006, USA	Retrospective	Reoperation vs. no surgery	136 (91 vs. 45)	Median 31	7	After recurrence
Schulick et al. (26)	Not reported, USA	Retrospective	Reoperation	47	Median 28	7	After reoperation
Zhang et al. (31)	2009–2020, China	Retrospective	Reoperation vs. no surgery	47 (21 vs. 26)	Median 25	8	After recurrence
Pommier et al. (30)	1980–1991, USA	Retrospective	Reoperation vs. no surgery	45 (26 vs. 19)	Median 28	7	Overall

### Prognostic Factors for Recurrent ACC

**Figure 2:**
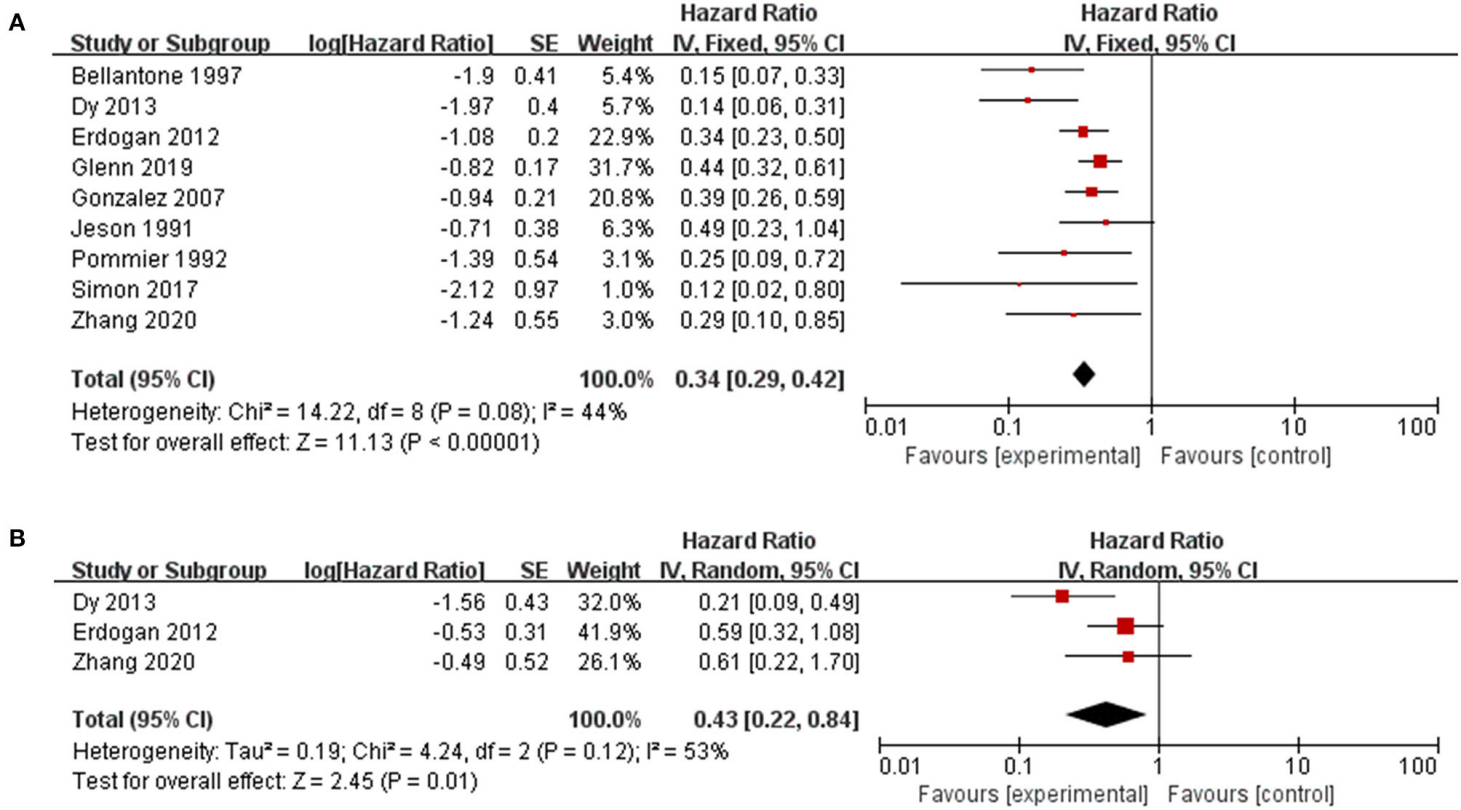
Pooled results of reoperation for recurrent ACC. **(A)** reoperation vs. nonoperative management; **(B)** debulking or incomplete resection vs. nonoperative management.

**Figure 3:**
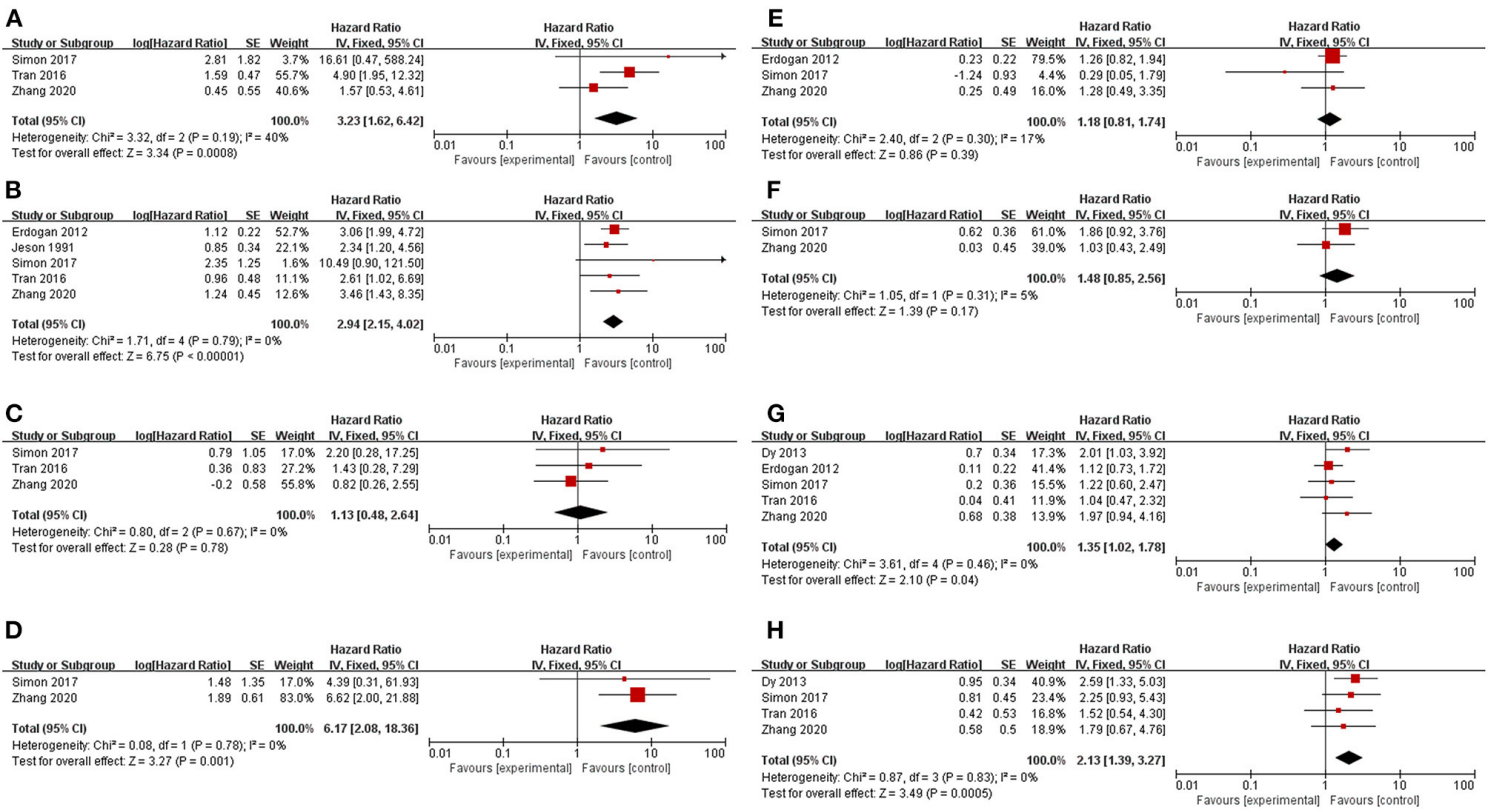
Prognostic factors for recurrent ACC patients. **(A)** Multiple recurrence; **(B)** Disease-free interval < 12 months; **(C)** Relatively high Ki67 index; **(D)** Stage III–IV of original tumor; **(E)** Age; **(F)** Relatively large original tumor size; **(G)** Sex (male vs. female); **(H)** Initial R0 resection.

Patients who underwent resection for recurrence had significantly better OS or OS after recurrence than those who received only nonsurgical treatments (HR 0.34, 95% CI 0.29–0.42, I^2^ = 44%, Figure 2). Factors were identified as prognostic factors associated with decreased OS after recurrence (Figure 3), including multiple recurrence (HR 3.23, 95% CI 1.62–6.42, I^2^ = 40%), shorter DFI (HR 2.94, 95% CI 2.15–4.02, I^2^ = 0), stage III–IV of original tumor (HR 6.17, 95% CI 2.08–18.36, I^2^ = 0), sex (HR 1.35, 95% CI 1.02–1.78, I^2^ = 0), and initial non-R0 resection (HR 2.13, 95% CI 1.39–3.27, I^2^ = 0). However, Ki67 (HR 1.13, 95% CI 0.48–2.64, I^2^ = 0), age (HR 1.18, 95% CI 0.81–1.74, I^2^ = 17%), original tumor size (HR 1.48, 95% CI 0.85–2.56, I^2^ = 5%), and adjuvant therapy (HR 0.92, 95% CI 0.57–1.49, I^2^ = 0) were not significantly related to OS after recurrence. In addition, three studies evaluated the effect of debulking or incomplete resection of recurrent ACC (Figure 2), and compared with patients receiving only nonsurgical treatments, a prolonged OS after recurrence was observed in those who experienced incomplete resection (HR 0.43, 95% CI 0.31–0.52, I^2^ = 53%).

### Identify Patients Who Benefit More From Reoperation

**Figure 4:**
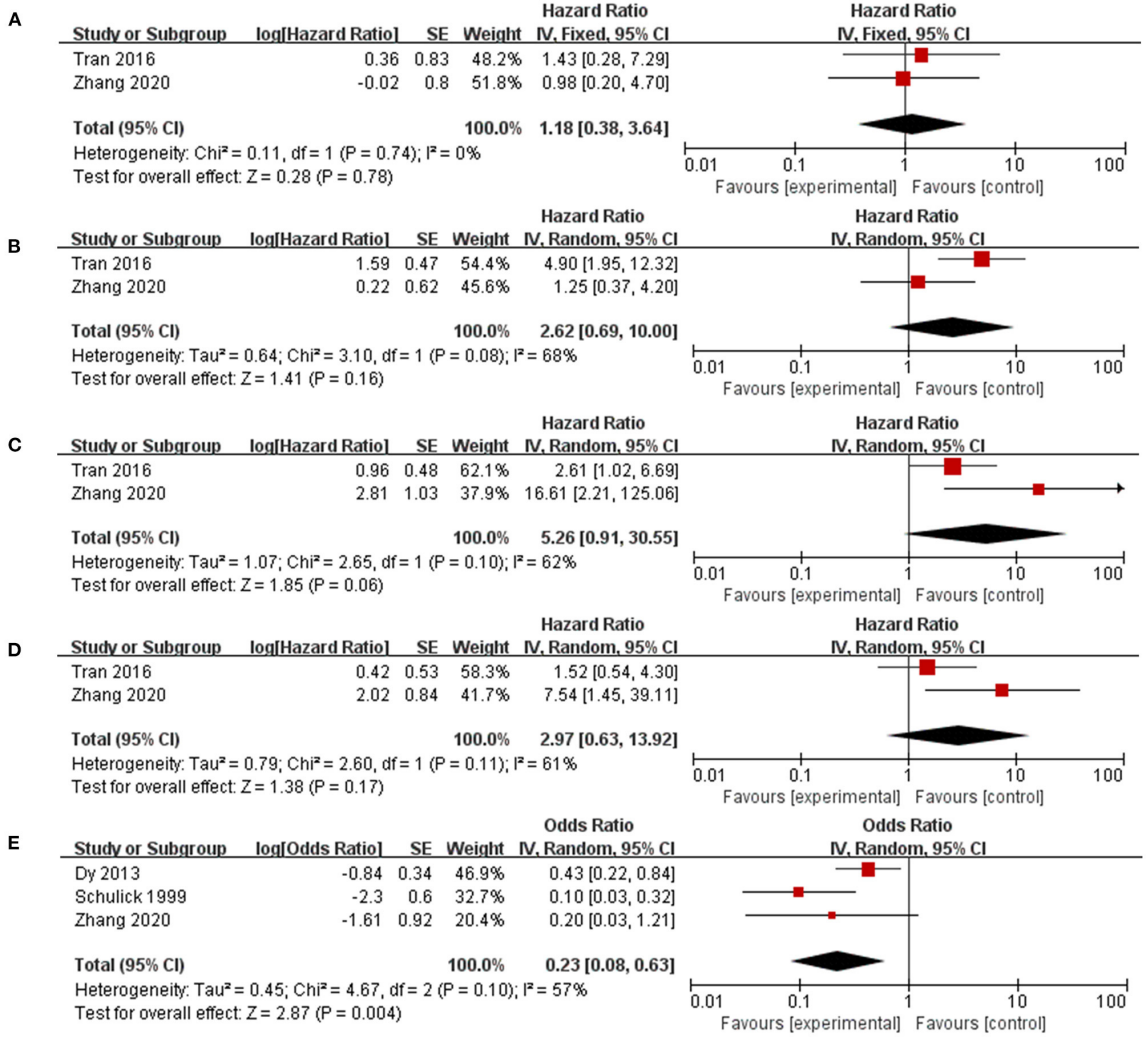
Factors that identify patients who benefit more from reoperation. **(A)** Relatively high Ki67 index; **(B)** Multiple recurrence; **(C)** Disease-free interval < 12 months; **(D)** Initial R0 resection; **(E)** Complete resection of recurrent ACC.

Patients with repeated surgeries were grouped by different parameters in five studies to identify potential prognostic factors related to a better prognosis (Figure 4). Patients with multiple recurrence (HR 0.2.62, 95% CI 0.69–10.00, I^2^ = 68%), shorter DFI (HR 5.26, 95% CI 0.91–30.55, I^2^ = 62%), and noninitial R0 resection (HR 2.97, 95% CI 0.63–13.92, I^2^ = 61%) tended to benefit less from reoperation, but the results were not significantly different. Furthermore, those who experienced complete resection of recurrent tumors (HR 0.23, 95% CI 0.08–0.63, I^2^ = 0) were associated with a significantly better OS after recurrence.

### Subgrou*p* Analysis

Subgrou*p* analyses were conducted based on the region (America, Europe), OS definition (OS after recurrence, OS calculated from the first diagnosis), and the number of patients (<60 and ≥ 60). In all subgroups, patients who underwent reoperation after recurrence were observed to have better OS or OS after recurrence, with all *p* ≤ 0.001 (Table 2).

**Table 2:** Subgroup analysis of reoperation for recurrent ACC based on region, definition of overall survival and number of included patients.

**Variable**	**No. of studies**	**Model**	**HR (95% CI)**	**p value**	**I^**2**^ (%)**
Total	9	Fixed	0.34 (0.29–0.42)	<0.001	44
Region					
America	5	Random	0.35 (0.24–0.50)	<0.001	51
Europe	3	Fixed	0.28 (0.20–0.40)	<0.001	50
OS definition					
OS after recurrence	6	Fixed	0.33 (0.26–0.42)	<0.001	34
OS	3	Random	0.27 (0.13–0.57)	0.001	69
No. of patients					
≥60	4	Random	0.34 (0.24–0.48)	<0.001	59
<60	5	Fixed	0.27 (0.17–0.41)	<0.001	24

### Sensitivity Analysis and Publication Bias

**Figure 5:**
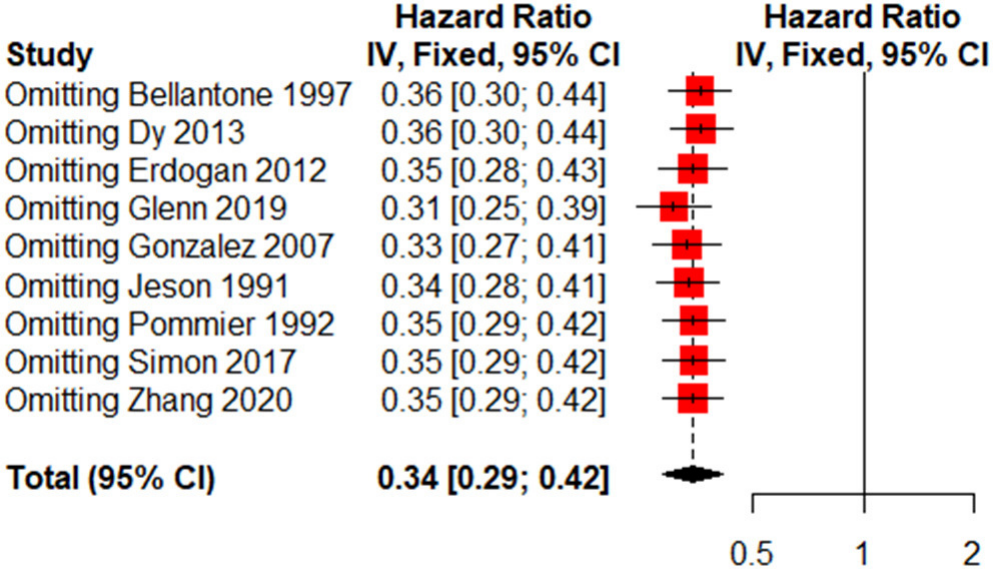
Sensitivity analysis of reoperation for recurrent ACC.

Due to the small number of included studies, we only performed sensitivity analysis and publication bias for reoperation. The sensitivity analysis is presented in Figure 5, and no evidence of publication bias was observed based on Begg's test (*p* = 0.180).

## Discussion

Adrenocortical carcinoma (ACC) is an aggressive tumor with a high recurrence rate. The current effective treatments for recurrent ACC are still limited. ACC is relatively resistant to chemotherapy and radiotherapy. Mitotane is the only effective drug approved by the FDA that has a temporary antitumor effect on ACC. However, this drug is limited by a very moderate response rate, a narrow therapeutic window, and a non-eligible rate of patients experiencing significant side effects (32, 33). The limitations of chemotherapy and radiotherapy for recurrent ACC patients highlight the importance of reoperation, which is regarded as a very aggressive approach but as the only curative treatment.

This study found that prolonged OS after recurrence was significantly related to reoperation in recurrent ACC patients, indicating that repeated surgeries are feasible for ACC recurrence. Furthermore, reoperation for ACC recurrence is also a relatively safe option in selected patients, and the mortality and morbidity in previous studies remained accepted. Simon et al. reported that thirty-day mortality was 3% (7), while Tran et al. reported thirty-day mortality of 5.4%, and serious complications were found in 18.6% of patients (15). We also evaluated the role of reoperation by subgrou*p* analysis, and the OS and OS after recurrence were significantly different between the two groups irrespective of region or cohort number.

Despite the fact that repeat surgeries were associated with better OS after recurrence, we noticed an inevitable selection bias for the reoperation inclusion criteria. In three studies (7, 28, 31) comparing the clinical and tumor characteristics of the two groups (reoperated vs. nonreoperated), those patients who underwent reoperation tended to have relatively smaller tumors, relatively shorter DFIs, a lower frequency of stage III-IV tumors, a single recurrence site, and a lower possibility of distant metastasis. This observation can be explained by tumors with these characteristics often being evaluated as an easy to complete resection during preoperative evaluation. Our study found that patients experiencing complete resection of recurrent lesions could benefit more from reoperation. Therefore, to select suitable patients for reoperation, it is extremely important to evaluate the possibility of complete resection of the lesion preoperatively. Apart from complete resection, there is a tendency that recurrent ACC patients with a single recurrence site, relatively longer DFI, and initial R0 resection would benefit more from reoperation. Consequently, we recommend that reoperation should be given priority in patients with these characteristics.

Interestingly, the pooled results showed that recurrent ACC patients benefited from prolonged survival even when they only received debulking or incomplete surgery. It is still controversial whether repeated surgeries should be performed in patients with advanced disease. Current guidelines do not recommend surgery for patients with multiple metastases. However, our study found that patients with advanced or multiple recurrent diseases might not be contraindicated for debulking or incomplete surgeries. Selected patients might benefit from a decrease in tumor volume, but this result could be restricted by the number of samples or some selection bias, and more cases should be accumulated and would be of great value.

In addition to reoperation, we identified several prognostic factors associated with OS after recurrence. The characteristics of tumors in primary resection are associated with survival when patients undergo recurrence. Initial R0 resection and tumor stage I-II are good prognostic signs that indicate that combat against recurrent ACC has already begun at the initial resection. A systematic meta-analysis conducted by Hu et al. reported that minimally invasive adrenalectomy surgery approaches were associated with earlier recurrence and more positive surgical margins and peritoneal recurrence than open adrenalectomy (5). Due to the fragility of ACC, it is essential for surgeons to carefully choose an appropriate surgical technique and achieve R0 resection of the primary tumor.

Another important prognostic factor for recurrent ACC patients is the length of time between initial resection and the first diagnosis of recurrence. A relatively shorter DFI, usually with a cutoff value of 12 months, is an independent prognostic feature predicting a poor prognosis. Winifred et al. showed that the DFI was significantly different between patients surviving >24 months after recurrence and patients surviving <12 months (34). In our opinion, DFI is an indicator of tumor characteristics and malignancy, which is similar to Ki67. The prognostic role of Ki67 in adrenocortical carcinoma after primary resection has already been reported by several studies (35, 36). However, in our study, no significant association was found between Ki67 and OS after recurrence. It might be partially explained that Ki67 could be used for stratifying patients with rapid recurrence, which causes a high rate of death risk and relatively shorter OS. Once the tumor recurs, this indicator is no longer significantly related to OS.

Another remarkable finding was that OS after recurrence was influenced by sex. Male patients had a worse prognosis after tumor recurrence. To the best of our knowledge, the association between sex and the prognosis of recurrent ACC has never been reported. This difference between sex and tumor mortality has been observed in many tumors; for example, men with breast cancer face high mortality rates (37). The exact mechanisms by which male patients might have a worse prognosis remain unknown and require further research.

Our research is not without obvious limitations. First, we only included 11 studies and 964 recurrent ACC patients, which is a relatively small number and may limit the effectiveness of the results to some extent. Next, all studies were retrospective and had an inherent bias, which may have influenced our results and partially resulted in heterogeneity between studies. To minimize this bias, we performed sensitivity and subgrou*p* analysis to further evaluate the stability of the included results. In addition, some factors may impact the results, including adjuvant therapy, follow-u*p* time, different treatment protocols from different institutions, etc. We performed a prior selection of multivariate analysis results instead of univariate analysis to minimize the influence of other factors. Furthermore, the time range of patients included was extensive from 1965 to 2020, which may affect our results due to the development and variation of surgical techniques and medical treatments for recurrent ACC.

## Conclusions

Our results confirm the role of reoperation in the clinical management of recurrent ACC. Select patients might benefit from debulking surgery. Preoperative evaluation of the complete resection of the recurrence is the key point to decide whether patients should undergo surgery. Furthermore, other prognostic factors associated with prolonged OS after recurrence include a single recurrence site, relatively longer DFI, stage I-II of the original tumor, and sex.

## Data Availability Statement

The original contributions presented in the study are included in the article/supplementary material, further inquiries can be directed to the corresponding authors.

## Author Contributions

FaZ, ZL, and DF wrote the main manuscript text. The data were collected by FaZ and DF and assessed by FaZ and ZL. Study was designed by YZ and YL. The figures and tables were prepared by FuZ, YT, and SL. All authors reviewed the manuscript, contributed to the article, and approved the submitted version.

## Funding

This work was supported by 1.3.5 Project for Disciplines of Excellence-Clinical Research Incubation Project, West China Hospital, Sichuan University.

## Conflict of Interest

The authors declare that the research was conducted in the absence of any commercial or financial relationships that could be construed as a potential conflict of interest.

## Publisher's Note

All claims expressed in this article are solely those of the authors and do not necessarily represent those of their affiliated organizations, or those of the publisher, the editors and the reviewers. Any product that may be evaluated in this article, or claim that may be made by its manufacturer, is not guaranteed or endorsed by the publisher.
